# YOU HAVE TO BE STRONG TO GET OLD: MIDDLE-AGE CAREGIVING, WISDOM DEVELOPMENT, AND LIFE PLANNING AMONG OLDER ADULT WOMEN

**DOI:** 10.1093/geroni/igz038.2701

**Published:** 2019-11-08

**Authors:** Kathryn Hartikka

**Affiliations:** University of Florida, Gainesville, Florida, United States

## Abstract

This study explores women’s development of wisdom in older adulthood resulting from caregiving of aging parents and spouses during their middle age years. As a way to grasp the complexity of middle age care work influencing wisdom development in older adulthood, this study used in-depth case analysis with in-depth interviews of three older adult women from the Silent Generation, ranging in birth years from 1933 to 1944. Women self-identified wisdom through themes such as compassion, knowledge attainment, and resilience resulting from their care work. This study also captured the significance of their caregiving in middle age as a turning point in their lives, marking their physical health and capabilities as notably stronger compared to their present selves in older adulthood. These women also illustrated increased awareness of their own mortality through caregiving. Jointly, these women self-identified their roles as caregivers as uniquely female roles. While all noted being confronted with care work as unexpected in their life path, these women believed in having a natural inclination to nurture and care, coupled with social pressures, leading to their ultimate caregiving. This study unearthed deeper meanings these women had for their lives resulting from caregiving, manifesting as knowledge gained about life, health, and aging. Thus, inspiring their own decision-making as they approach even older adulthood experiences such as dependency and death. This study ultimately illustrates the importance of caregiving on women’s lives: marking their middle age, increasing their wisdom development, and influencing their life planning as they age.

